# Perforated Gastric Body Secondary to Migrated Esophageal Stent

**DOI:** 10.7759/cureus.14314

**Published:** 2021-04-05

**Authors:** Clyde M Stauffer, Mohan Kulkarni

**Affiliations:** 1 General Surgery, Henry Ford Health System, Jackson, USA; 2 Thoracic Surgery, Henry Ford Health System, Jackson, USA

**Keywords:** esophageal stricture, gastric perforation, esophageal stent migration, perforated viscus, surgical emergency

## Abstract

A 44-year-old male presented to the ER with severe abdominal pain and a reported syncope episode. Focused abdominal sonography in trauma and abdominal exam demonstrated free fluid and peritonitis. CT scan demonstrated a metallic object in the stomach with free air and fluid throughout the abdomen. The following report is a rare case presentation of a perforated stomach secondary to a migrated esophageal stent used for the treatment of a benign esophageal stricture refractory to other treatment modalities.

## Introduction

Esophageal stents are utilized for many different purposes, including treatment of benign or malignant esophageal disease such as small, contained esophageal perforations, strictures, and unresectable malignancies. This also includes progressive chronic dysphagia that has failed medical management and other conservative measures. In regard to chronic dysphagia, the decision for stent placement is decided after serial dilations and failed medical management. Refractory benign esophageal strictures (RBES) can be defined as stenosis <14 mm after serial esophageal dilations within two weeks or failure to maintain a diameter >14 mm after four weeks of esophageal dilations [1,2]. Stents can be made of metal or plastic and can be self-expanding, covered, partially covered, or non-covered. Stents are often referred to as self-expanding metal stents (SEMS) or self-expanding plastic stents (SEPS). A non-covered stent can cause tissue erosion and can be difficult to remove. Secondary to this, they are rarely used today. Covered stents are better tolerated and can be easier to remove; however, they are more prone to migration. Partially covered stents have a small portion of metal (or plastic) exposed at each end of the stent, and this helps to prevent migration of the stent [3]. In general, stents should extend at minimum 1 cm proximal and distal to a given stricture or mass for appropriate therapeutic effect. Failure to provide these margins can result in ineffective treatment and continued dysphagia [4].

## Case presentation

**Figure 1:**
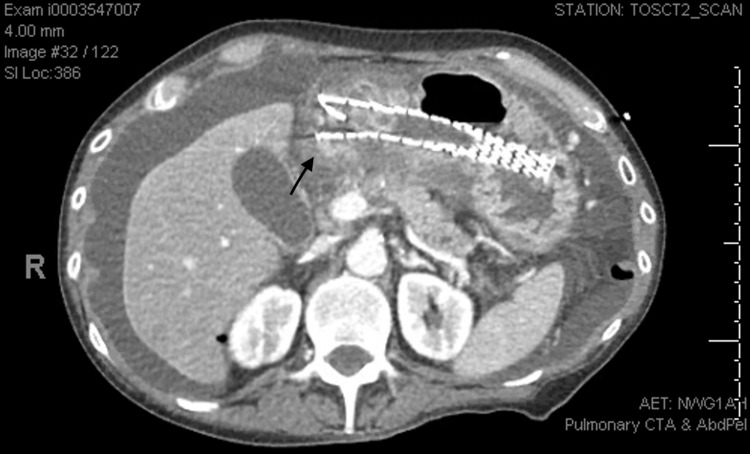
Metallic stent located in the gastric lumen with the tip appearing at the edge of the gastric wall (black arrow), as well as intra-abdominal free fluid throughout the abdomen.

A 44-year-old chronically unhealthy male with a history of alcohol abuse, gastroesophageal reflux disease (GERD), hypertension, esophageal stricture, and dysphagia was previously seen by thoracic surgery for evaluation of weight loss and continued difficulty swallowing. He had previously undergone several esophagogastroduodenoscopies (EGDs) with serial dilations and only mildly improved dysphagia. No reports of peptic ulcer disease (PUD) were identified during previous EGDs. Images and biopsies were negative for esophageal malignancy or mass but did demonstrate severe stenosis. Due to recurrent symptoms and progressive dysphagia resulting in weight loss and the inability to swallow liquids and medications, he was treated with repeat esophageal dilation and placement of a 19 mm x 120 mm fully covered EndoMAXX esophageal stent by Merit Medical Systems. During outpatient follow-up, four weeks later, he was progressing well and able to swallow oral liquids and solids. The patient reported a 20 lb weight gain since stent placement and was pleased with his post-operative progression. Five months later, the patient presented to the ED following a syncope episode and acute onset abdominal pain. In the ER, due to the uncertain presentation, he was treated as a trauma patient. He underwent workup with a Focused Abdominal Sonography in Trauma (FAST) exam, which demonstrated intra-abdominal free fluid. Physical examination demonstrated severe tenderness consistent with peritonitis, as well as transient hypotension that responded to IV resuscitation. A CT scan of the abdomen and pelvis was obtained and demonstrated free fluid, air, and an intra-abdominal metallic object (Figure 1). Based on findings and physical exam, the patient was transported to the operating room for an emergent exploratory laparotomy. Intra-operative findings consisted of gastric perforation in the body of the stomach with what appeared to be an esophageal stent protruding through the gastric wall. At this time, the perforation was extended, and the stent was removed. The gastric perforation was closed primarily with a tongue of omentum placed over the incision (pedicled omentoplasty). The patient's hospital course was complicated with a post-operative stroke and eventual mortality.

## Discussion

Esophageal strictures are caused by many different pathologies, including GERD, radiation, cancer, esophageal resection, immunologic disorders, and more. The disease can be very aggravating and debilitating for affected individuals. Typically, about two in three patients will be successfully treated with serial dilations. However, one-third may require further management. Complex strictures are defined as being longer than 2 cm, tortuous, and severely narrowed diameter [5]. In this patient, he had a complex medical history that included alcohol abuse and chronic reflux that, when able to swallow medications, was effectively treated. Secondary to his inability to swallow and recurrent stricture formation following dilation, it was concluded that he would benefit from stent placement to better assist in oral intake and compliance with medication regimens. The placement of esophageal stents should be thoroughly discussed with the patient before placement. The decision to proceed with stent placement should be held if serial dilations, injections, and or cutting dilations have failed to treat the stricture appropriately. Once decided to proceed, an EGD is performed, a wire is placed in the stomach for guidance, and fluoroscopy is used to ensure the stent is deployed at the appropriate junction [4]. The stent should extend 1 cm above and below the stricture to ensure coverage and therapy appropriately. It is not necessary to dilate the stricture before placing the stent; however, if the passage of the stent device proves difficult, dilation can be performed [4]. Complications of stent placement are not benign and can be severe, although rare. These include esophageal perforation following deployment, reactions to the stent material, worsening dysphagia, obstruction, and migration. As previously discussed, stents can be covered, non-covered, and partially covered. Partially covered are more often selected for therapy as they provide appropriate treatment, have decreased esophageal ingrowth, and have a lesser chance of migration [3]. A review of the literature does demonstrate that stent migration can occur and cause intestinal obstruction; however, this is rare, and only a small number of cases have been reported. Therapy for stent migration into the small intestine, causing obstruction, does require surgical removal, as it is highly unlikely to resolve with conservative management. Besides, without the removal of an obstructing stent, it will result in a small bowel perforation likely secondary to distention and eventual erosion into the bowel wall [6]. In non-covered and partially covered stents, there are two forces generally used to help prevent stent migration. The expansive force of the stent pressing against the esophageal wall and the second being tissue ingrowth into the open component of the stent. In theory, if the stent is appropriately deployed, and the tissue appropriately grows into the stent material, the stent should not migrate from its fixed position, and most of the time, it does not. Stent complications can be defined as early and delayed. Hindy et al. define these terms as early being immediately or up to four weeks post-procedure and delayed being any time after four weeks. Early complications can occur in up to 32% of patients with migration as the most significant complaint. During the early post-procedure period, the stent positioning mainly relies on the expansive forces of the stent, thus making this period more likely to have stent migration. Delayed complications of stent migration have been reported around 9% [3]. In this patient, it is uncertain what caused his stent to migrate after five months of successful therapy and or what caused the gastric perforation. It is rare for a stent to perforate the intestine, but even more rare for it to cause a perforation in the stomach body, as the stomach can expand to accommodate the size of the stent. During the initial exploratory laparotomy, the tissue was severely inflamed, and the intra-abdominal cavity did contain significant amounts of food and gastric fluids. With speculation, the stent may have migrated much earlier than the day he had presented to the hospital, and the patient had been experiencing nausea, abdominal pain, and dysphagia for several days, but failed to seek treatment. The syncope episode could have been from severe sepsis and thus provided objectifiable symptoms seen by family members that indicated emergency transportation and evaluation. Examination of the stomach during the gastrostomy did not reveal any signs of gastric ulcers, that if present, would have compromised the integrity of the stomach and increase the chances of accidental perforation. A review of the literature available did not demonstrate any previous cases of stomach perforation secondary to stent migration. While there are few cases available regarding stent migration and small bowel obstruction/perforation, gastric perforation is very rare [6].

## Conclusions

Esophageal stents are an essential tool that has been shown to treat dysphagia successfully, secondary to refractory esophageal structures. Migration is a complication that is more likely to occur early following placement but can occur at a later date, although rare. Intestinal obstruction is more commonly diagnosed following evaluation of a migrated esophageal stent. Intestinal perforation is possible but rare. Perforation of the stomach is extremely rare, and no identified case reports have been identified during the literature review to formulate this report. As with any gastrointestinal perforation, if not treated quickly, it can result in severe sepsis and patient demise.
